# The whole genome sequencing offers insights into the susceptibility to the foot-and-mouth disease virus carrier state

**DOI:** 10.1186/s13567-025-01697-4

**Published:** 2026-01-03

**Authors:** Zhihui Zhang, Zhidong Teng, Shuanghui Yin, Suyu Mu, Sumin Wei, Yaozhong Ding, Yun Zhang, Shuang Wang, Yijing Li, Shiqi Sun, Huichen Guo

**Affiliations:** 1 State Key Laboratory for Animal Disease Control and Prevention, College of Veterinary Medicine, Lanzhou University, Lanzhou Veterinary Research Institute, Chinese Academy of Agricultural Sciences, Lanzhou, 730046 China; 2 College of Veterinary Medicine, Northeast Agricultural University, Harbin, 150030 China; 3 College of Veterinary Medicine, Gansu Agricultural University, Lanzhou, 730070 China; 4 Yunnan Tropical and Subtropical Animal Virus Diseases Laboratory, Yunnan Animal Science and Veterinary Institute, Kunming, 650225 China; 5 School of Life Sciences, Ningxia University, Yinchuan, 750021 China

**Keywords:** Foot-and-mouth disease virus, viral persistence, genetic variations, disease susceptibility, SNPs

## Abstract

Foot-and-mouth disease virus (FMDV) establishes persistent infection in more than 50% of infected ruminants, irrespective of vaccination status, implicating potential contributions of host genetic variations to viral persistence. In this study, we conducted whole-genome resequencing of a cohort of 22 cattle, comprising 7 carriers and 15 noncarriers. Clean reads were mapped to the cattle (*Bos taurus*) reference genome (ARS-USD1.2) and cattle–yak (*Bos taurus* × *Bos grunniens*) haplotype assemblies. We identified 24 shared carrier-specific variants across genomes and 31 carrier-specific variants restricted to the cattle–yak haplotypes. These carrier‑specific variants were primarily located in genes involved in olfactory perception, cell development and morphological maintenance, transcriptional and translational regulation, signal transduction, metabolic homeostasis, stress resistance, and immune cell regulation. The combined functional impact of these variants may influence innate immune regulatory capacity and shape the magnitude of adaptive immune activation, thereby conferring distinct antiviral capacities and facilitating viral persistence. Our findings offer a comprehensive catalog of candidate genetic variants potentially associated with persistent FMDV infection, providing novel insights into the genetic architecture underlying host's susceptibility to viral persistence.

**Supplementary Information:**

The online version contains supplementary material available at 10.1186/s13567-025-01697-4.

## Introduction

Foot-and-mouth disease (FMD) is a highly contagious and economically devastating viral disease of livestock, caused by infection with FMD virus (FMDV), an *Aphthovirus* within the *Picornaviridae* family. The disease affects more than 70 species of wild and domestic cloven-hoofed animals, including major livestock species such as cattle, pigs, sheep, and goats, as well as buffalo and wild boar [1]. In FMD-endemic regions, substantial annual losses arise from vaccination costs, enforcement of animal health measures, and trade restrictions [2, 3]. Conversely, FMD-free countries invest heavily in preparedness and surveillance, as any outbreak could severely disrupt livestock production and animal product trade [4]. FMD presents exceptional challenges to control owing to its minimum infectious dose, rapid viral replication and broad host range, which is further compounded by subclinical persistent infection in ruminants following viral exposure [5]. The persistent FMDV infection, also known as the FMDV carrier status, is defined as the recovery of the virus from oropharyngeal fluid (OPF) more than 28 days post-infection by the World Organization for Animal Health (WOAH) [6].

Clinical investigations have documented an incidence exceeding 50% for FMDV persistence, even among herds fully protected by vaccination [7–9]. The duration of the carrier state varies among animal species, lasting up to 9 months in sheep, 3.5 years in cattle, and 5 years in African buffalo [10]. The anatomical location of viral persistence also varies among animal species. In cattle, FMDV is primarily confined to the nasopharynx [7]. In sheep, it persists within the epithelial crypts of the oropharyngeal and laryngeal tonsils [11]. In African buffalo, viral persistence is observed in the pharyngeal and palatine tonsils, as well as the nasopharyngeal mucosa [12]. Critically, experimental data demonstrate that transmission from carriers occurs [13–16]. Although the individual transmission probability appears minimal [17], the high prevalence of FMDV infection and frequent cross-border animal movements amplify the number of carriers and heighten population-level epidemiological concerns. Moreover, heterologous FMDV superinfection in carrier cattle frequently drives viral recombination in the upper respiratory tract, generating dominant interserotype recombinants [18, 19]. The shedding of such recombinant viruses may lead to subclinical infections without overt clinical manifestations. These epidemiological and virological factors make carrier animals a significant barrier to FMD control, eradication, and attainment of WOAH FMD-free status.

The viral genetic determinants of FMDV persistence have been extensively investigated, yet most studies have failed to identify specific genomic features correlated with the carrier state [20–23]. Although particular type O strains exhibit specific amino acid substitutions, such as VP1 Q172R (ME-SA/PanAsia) [24], VP2 Y80H (UKG34/2001) [25], VP2 Y79H and VP3 A75T (FRA/1/2001) [26], these associations lack further corroboration. Consistent with SAT1/KNP/196/91 strain in buffaloes [20], persistent isolates of O/FRA/1/2001 strain show no evidence of antigenic escape from neutralizing antibodies [26]. Litz et al. reported that leaderless O/FRA/1/2001 failed to cause clinical disease or persistence in cattle [27], though it is unclear whether similar effects occur across other strains or serotypes. While SAT1/KNP/196/91 strain exhibited greater prevalence and longer persistence than SAT2/KNP/19/89 and SAT3/KNP/1/08 strains in coinfected African buffalo [12], no competitive advantage was observed between O1 Manisa and A24 Cruzeiro strains in coinfected cattle [18, 28]. Collectively, these findings suggest that while viral determinants may contribute to persistence, host factors play a decisive role, as in vivo FMDV replication in cattle triggers strong antiviral responses [9, 29–31].

In carrier cattle, antiviral responses in microdissected FMDV-positive nasopharyngeal epithelium are locally suppressed, without obvious histopathological lesions [8], suggesting a localized virus–host equilibrium. Viral clearance is primarily driven by adaptive immunity, with FMDV infection and vaccination predominantly inducing robust antibody-mediated responses. However, systemic antibody titers did not differ between carriers and noncarriers [9, 29]. Although carriers exhibit stronger FMDV-specific IgA responses in nasal and oral secretions [32–34], they remain unable to eliminate the virus sequestered within host cells. Transcriptome analyses of microdissected nasopharyngeal epithelium have linked the carrier state to the impaired cell-mediated immunity [35, 36], as noncarriers exhibit higher T cell infiltration within nasopharyngeal mucosa [35], and cellular immune responsiveness of peripheral blood mononuclear cells inversely correlates with viral persistence [37]. Whether host-intrinsic factors govern clearance versus persistence remains unclear.

Interindividual genetic variations can influence both susceptibility to and clinical progression of viral infection. Polymorphisms in innate immune-related genes (*OAS1* [38], *IFNAR1* [39], *MxA* [40], and *IRTK4* [41]) and viral receptor genes (*SCARB2*, *PSGL1*, and *ANXA2*) [42] have been linked to the severity of enterovirus 71-induced hand-foot-and-mouth disease. Moreover, loss-of-function mutations of *IFIH1* increase vulnerability to respiratory RNA viruses, including respiratory syncytial virus and rhinoviruses [43]. Regarding FMDV, polymorphisms in the *BoLA-DRB3* gene [44, 45] or in integrin genes [46, 47] potentially influence disease susceptibility. Five *BoLA-DRB3* genotypes have been reported in Egyptian buffalo populations, with the AA genotype associated with FMD resistance and the AC genotype with greater FMD susceptibility [48]. Genome-wide association studies (GWAS) have demonstrated that resistance to FMDV in Holstein cattle is associated with enhanced innate immune responses [49]. Compared with Holstein–Friesian (*Bos taurus*) cattle, two Indian breeds–Malnad Gidda and Hallikar (*Bos indicus*)–showed a significant upregulation of genes involved in mitochondrial activation and innate antiviral immune pathways during acute infection, leading to reduced viral loads and earlier induction of neutralizing antibody and interferon-gamma (IFN-γ) responses [50]. Therefore, a comprehensive understanding of host genetic determinants underlying persistent FMDV infection informs virus–host coevolution studies and supports host-targeted strategies, including disease-resistance breeding and personalized modulation.

To this end, we conducted whole-genome sequencing (WGS) to identify differential genetic variations between vaccinated carriers and noncarriers. We propose a set of candidate variants potentially implicated in the establishment of persistent FMDV infection. Our findings provide novel insights into the genetic architecture underlying viral persistence and offer avenues for improved prevention and control strategies.

## Materials and methods

### Samples and sequencing

Peripheral blood was collected from 22 vaccinated cattle, comprising 7 FMDV carriers and 15 noncarriers. The detailed demographic and clinical characteristics of the enrolled subjects are provided in Additional file 1. Genomic DNA was extracted via the DNeasy Blood and Tissue Kit (Qiagen) following the manufacturer’s protocol. The concentration and quality of the extracted genomic DNA were assessed via a NanoDrop 2000 Spectrophotometer (Thermo Fisher Scientific). Sequencing libraries were prepared using the NEBNext DNA Library Prep Reagent Set (BioLabs, no. E6000). High-throughput sequencing was conducted on an Illumina NovaSeq 6000 platform (Illumina, CA, USA), generating 150-bp paired-end reads for downstream analyses.

### Reads alignment

To minimize artificial bias in sequencing reads, raw reads were filtered out according to the following criteria: (1) the content of unidentified nucleotides was greater than 10%; (2) reads with > 10 nucleotides aligned to the adapter, allowing ≤ 10% mismatches; and (3) average base-quality less than 20 (Phred-like score). Finally, the N Tb (Q30 = 90.0%) values of the high-quality sequences were obtained for subsequent analyses. The clean paired-end reads were then aligned to the cattle (*Bos taurus*) reference genome (ARS-USD1.2) via the maximum entropy method (MEM) algorithm of Burrows-Wheeler Aligner (BWA) software (v.0.7.8) with the optimized parameters (‘men -t 4 -k 32 -M’). The resulting alignment files in SAM format were processed via SAMtools (v.1.3) to convert, index, and sort the data, ultimately generating BAM files. If multiple read pairs had identical external coordinates, only the pair with the highest mapping quality was retained. Potential PCR duplicates were removed via Picard (v1.9.4) to improve alignment results.

To assess potential sex-related effects on the analysis, we retrieved the sex-determining region Y (*SRY*) gene sequences of cattle and yak from the Ensembl website. Clean reads were aligned to the *SRY* reference sequences. Substantial read coverage across multiple Y-specific regions was considered indicative of male individuals.

### SNPs/InDels calling and annotation

After alignment, single nucleotide polymorphisms (SNPs) and short insertions and deletions (InDels, 1–49 bp) for all individuals were called simultaneously using the Genome Analysis Toolkit (GATK) HaplotypeCaller (release 4.0). SNPs were filtered using the following criteria: variant quality by depth (QD) < 2.0; Phred-scaled *p*-value using Fisher’s exact test to detect strand bias (FS) > 60.0; root mean square of the mapping quality score (MQ) < 40.0; strand odds ratio of 2 × 2 contingency table to detect strand bias (SOR) > 3.0; mapping qualities of Ref reads (MQRankSum) < −12.5; and ranked-sum test for the distance of alleles from the end of the reads (ReadPosRankSum) < −8.0. We then filtered out allosomal SNPs and SNPs with a missing genotype rate > 0.1 and a minor allele frequency (MAF) < 0.05.

InDels were filtered using the following criteria: QD < 2.0; FS > 200.0; MQ < 40.0; SOR > 10.0; MQRankSum < −12.5; and ReadPosRankSum < −8.0. The autosomal InDels with a MAF ≥ 0.05 and a missing genotype rate of 0 were subsequently retained for further analyses.

### Identification of differential variants between carriers and noncarriers

Among all autosomal variants from 22 cattle retained after quality filtering, we selected missense variants within splice donor sites and exonic regions to identify differential variants that were polymorphic between carriers and noncarriers via Fisher’s exact test. Variants exhibiting extremely statistical significance (*p*-value < 0.01) were retained for subsequent downstream analyses.

### Allelic frequency differentiation between carriers and noncarriers

We performed fixation index (*F*_ST_) analysis using VCFtools (0.1.16) [51] to quantify allele frequency differentiation between carrier and noncarrier groups across autosomes, focusing on missense variants located within splice donor sites and exonic regions. The variants with top 1% *F*_ST_ values were considered to be of strong selective pressure or genetic divergence between the two groups and were subjected to further analyses.

### Determination of candidate regions or genes

The overlapping genomic regions, with both a *p*-value < 0.01 determined by Fisher’s exact test and a top 1% *F*_ST_ value, were subjected to gene annotation. Among overlapping regions, genes harboring variants exclusively observed in the carrier group were identified as candidate genes that differentiate carriers and noncarriers.

### GWAS

To identify significantly associated SNPs with the carrier state, the R package rMVP (version 1.4.5) was applied for case–control GWAS. Principal component analysis (PCA) was performed to estimate and adjust population stratification. Kinship matrices were generated to calculate the pairwise kinship coefficients required to correct for relatedness among individuals using the identity-by-state (IBS) method. GWAS analyses were conducted using generalized linear model (GLM), mixed linear model (MLM), and FarmCPU model simultaneously, with a threshold of 5 × 10^−8^, incorporating both PCA results and the kinship matrix to adjust for population structure and relatedness. Manhattan and Quantile–quantile plots were generated to visualize GWAS results and assess the overall fit of the model, which confirmed proper control of inflation factors typically affected by population stratification and kinship. Significant associations were identified on the basis of a genome-wide significance threshold corrected for multiple testing.

### Cattle–yak haplotype genome mapping

The cattle–yak (*Bos taurus* × *Bos grunniens*) haplotype 1 and haplotype 2 assemblies were generously provided by Professor Minghui Kang and Jianquan Liu, which remain unpublished to date. The clean reads were aligned to the cattle–yak (*Bos taurus* × *Bos grunniens*) haplotype 1 and 2 genomes separately. The missense variants within exonic regions and splice donor sites were selected to identify differential variants between carriers and noncarriers using Fisher’s exact test. The identified candidate variants based on the cattle (*Bos taurus*) reference genome (ARS-USD1.2) were mapped to these two haplotype genomes to assess potential artifacts arising from hybrid recombination.

## Results

### Genomic landscape of SNPs and InDels

A total of approximately 4.3 billion paired-end clean reads were generated from WGS of 22 cattle samples, with an average coverage depth of ~10× on the Illumina sequencing platform. These reads were aligned to the cattle (*Bos taurus*) reference genome (ARS-UCD1.2), attaining a high average genome coverage of 97.92% (Additional file 2). After quality filtering, we identified 32 883 797 autosomal SNPs with missing genotype rates < 0.1 and MAFs ≥ 0.05 (Additional file 3), and 2,834,320 InDels with missing genotype rates = 0 and MAFs ≥ 0.05 (Additional file 4). These high-quality variants were subjected to subsequent analyses.

**Figure 1:**
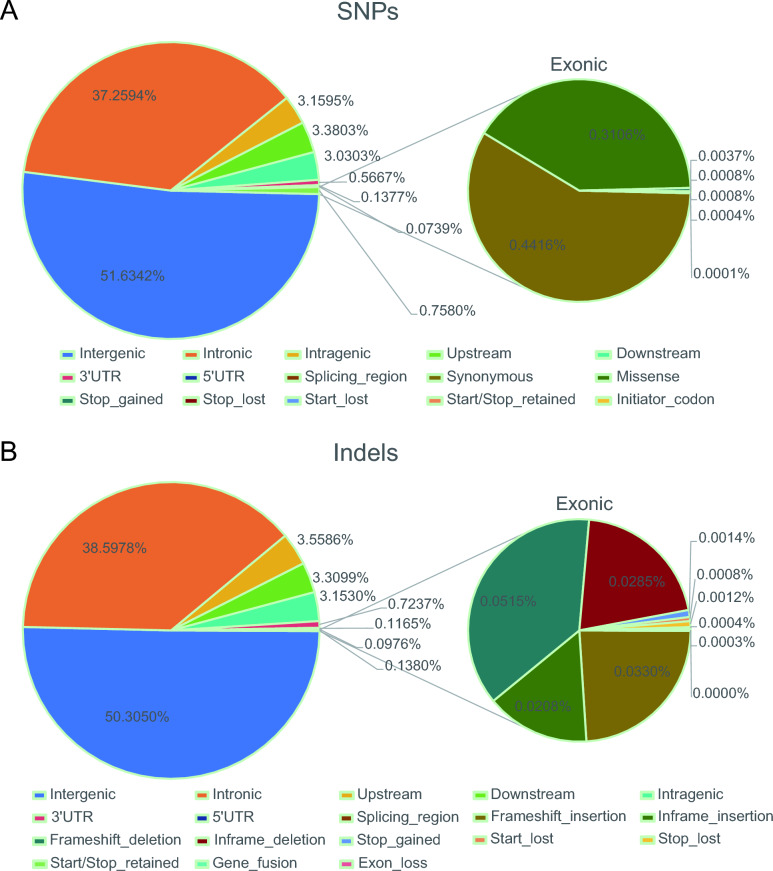
**Functional classification of the detected SNPs and InDels in genomes**. **A** Frequency of different functional types of the total SNPs. **B** Frequency of different functional types of the total InDels.

Functional annotation of retained SNPs revealed that the majority were situated in intergenic regions (51.63%) or intronic regions (37.30%). Exonic SNPs, which accounted for 0.76% of the total SNPs (Figure 1A), included 102,215 missense SNPs, 145,226 synonymous SNPs, 1213 stop-gained SNPs, 278 stop-lost SNPs, 253 start-lost SNPs, 131 start/stop-retained SNPs, and 22 initiator-codon SNPs (Additional file 3). For the InDels, functional annotation demonstrated that the majority were also located in intergenic regions (50.31%) or intronic regions (38.60%). Exonic InDels accounted for 0.14% of the total InDels (Figure 1B), comprising 935 frameshift-insertion InDels, 1460 frameshift-deletion InDels, 590 inframe-insertion InDels, 807 inframe-deletion InDels, 40 stop-gained InDels, 34 stop-lost InDels, 23 start-lost InDels, 11 start/stop-retained InDels, 9 gene-fusion InDels, and 1 exon-loss InDel (Additional file 4).

### Differential variants between carriers and noncarriers

To elucidate the genetic underpinnings of susceptibility to the FMDV carrier state, we focused on variants showing marked differences in allele frequency between carriers and noncarriers. These variants are hypothesized to play a critical role in the pathogenesis of persistent FMDV infection. Considering the potential influence of population structure on the analysis results, we performed principal component analysis (PCA) and kinship analysis. The results revealed no significant population stratification between carriers and noncarriers (Additional files 5A, 5B, 6). Subsequently, we performed a case–control (noncarrier–carrier) GWAS using the R package rMVP with GLM, MLM, and FarmCPU models simultaneously. GLM model identified six autosomal SNPs supposed to be significantly associated with the FMDV carrier state, with five SNPs in the intergenic regions and one in the intron (Additional files 7A, 7B, 8). Unexpectedly, only one locus (NC_037337.1:32701) was observed in carriers, which was more prevalent in noncarriers. Notably, no variants were found to be significantly associated with the carrier status in MLM and FarmCPU models. This outcome is highly probable owing to the inadequate sample size.

**Figure 2:**
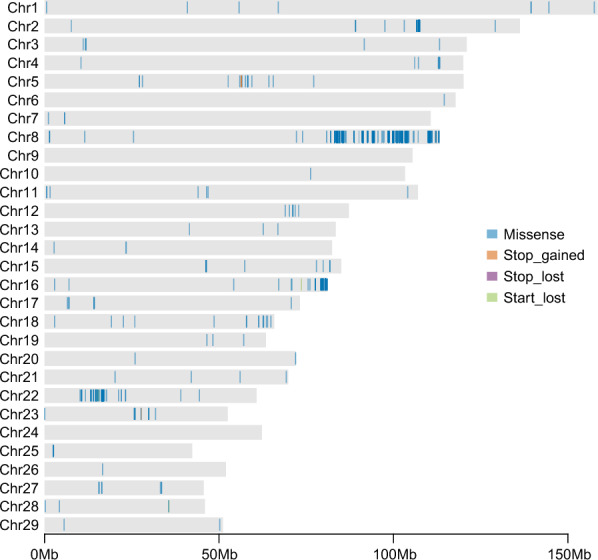
**Distribution of the differential SNPs (*p*-value < 0.01) between carrier and noncarrier groups identified by Fisher’s exact test.** The *x*-axis represents the chromosome positions and the *y*-axis indicates the chromosome number.

To address this, we applied Fisher’s exact test to identify variants exhibiting statistically significant differences in allele frequency between the two groups. We identified 532 exonic SNPs showing extremely significant differences in allele frequency between carriers and noncarriers (*p-*value < 0.01). These SNPs were located within 286 genes, including 523 missense SNPs in 278 genes, 4 start-lost SNPs in 4 genes, 4 stop-gained SNPs in 3 genes, and 1 stop-lost SNP in 1 gene (Additional file 9). Notably, the majority of these differential SNPs were located predominantly on chromosomes 8, 16, and 22 (Figure 2).

**Figure 3:**
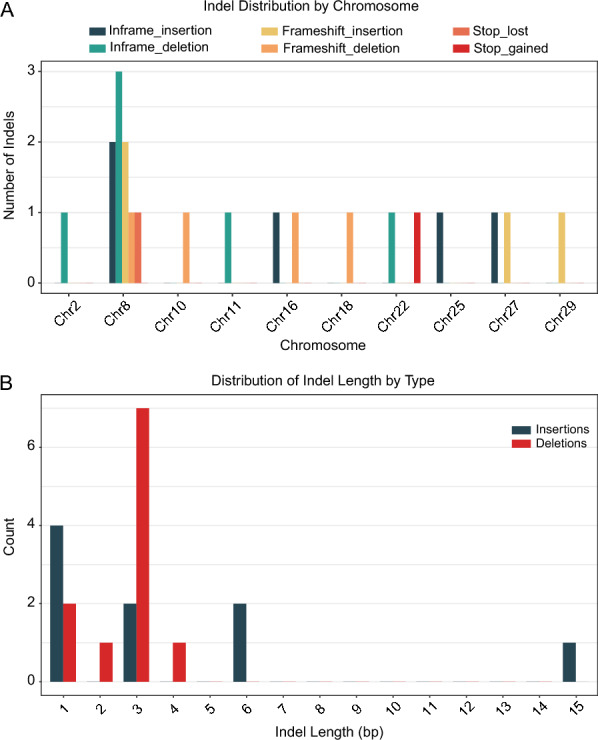
**Distribution of differential InDels (*p*-value < 0.01) between carrier and noncarrier groups identified by Fisher’s exact test.** **A** The number of differential InDels across chromosomes. **B** Distribution of differential InDels length (bp).

Concurrently, we identified 22 extrenely differential exonic InDels (*p*-value < 0.01) between carriers and noncarriers that covered 21 genes, including 7 inframe-deletion InDels within 7 genes, 5 i-frame-insertion InDels within 5 genes, 4 frameshift-deletion InDels within 4 genes, 4 frameshift-insertion InDels within 4 genes, 1 stop-gained InDel within 1 gene, and 1 stop-lost InDel within 1 gene (Additional file 10). These differential InDels were distributed mainly on chromosomes 2, 8, 10, 11, 16, 18, 22, 25, 27, and 29 (Figure 3A). Among them, the largest one was a 15-bp insertion, while the majority ranged from 1 to 3 bp (Figure 3B).

### Variants with highly allelic frequency differentiation between carriers and noncarriers

**Figure 4:**
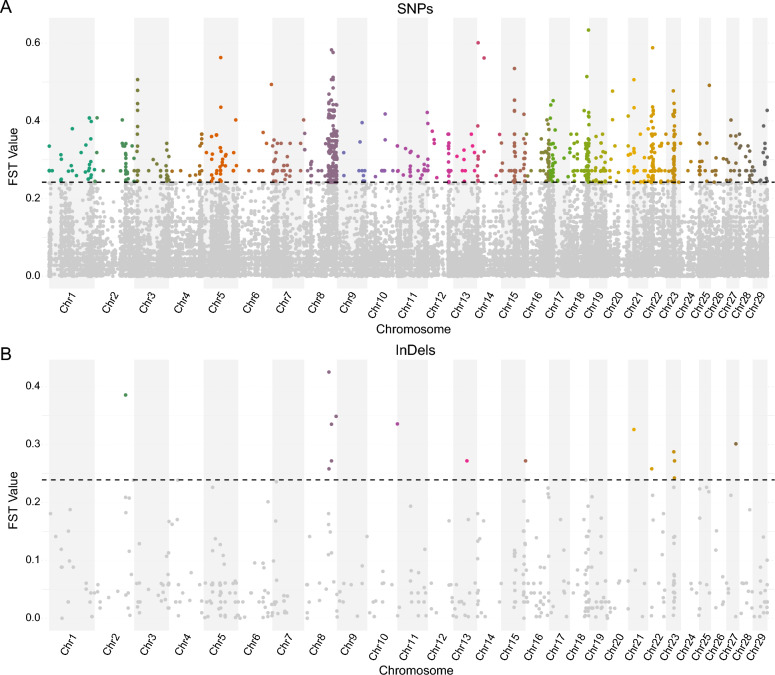
**Genome-wide scans for signatures of selection between carrier and noncarrier groups**. **A** Manhattan plot for the *F*_ST_ values (*y*-axis) of selected autosomal SNPs across all autosomes (*x*-axis). **B** Manhattan plot for the *F*_ST_ values (*y*-axis) of selected autosomal InDels across all autosomes (*x*-axis). The dashed lines represent thresholds for the top 1% *F*_ST_ values.

We then used the fixation index (*F*_ST_) method to quantify each missense variant across autosomal exonic regions and splicing donor sites to identify variants with extreme allele frequency differentiation between carrier and noncarrier groups. On the basis of the top 1% of the *F*_ST_ values, we identified 1063 SNPs covering 660 genes (*F*_ST_ ≥ 0.242161, Figure 4A; Additional file 11) and 15 InDels covering 14 genes (*F*_ST_ ≥ 0.257986, Figure 4B; Additional file 12). These variants represent the most significant differences in allele frequency between carrier and noncarrier groups.

### Candidate variants associated with the carrier state

**Figure 5:**
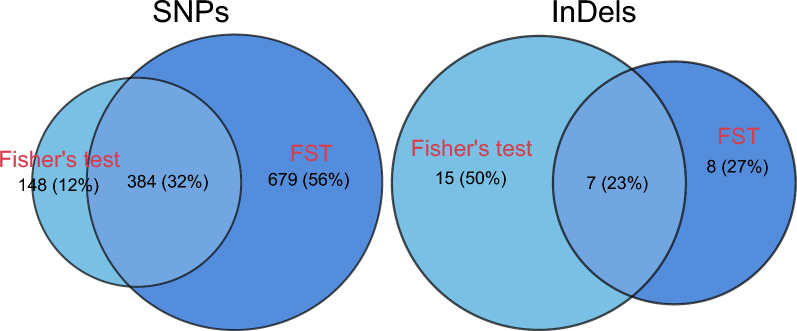
**Identification of the overlapping variants**. Venn diagram showing the overlapping region counts of SNPs (left) and InDels (right) obtained based on Fisher’s exact test and *F*_ST_ methods.

To confirm genetic variations significantly associated with persistent FMDV infection, we intersected genomic regions meeting two stringent criteria: (1) *p*-values < 0.01 based on Fisher’s exact test, and (2) *F*_ST_ values within the top 1% of the distribution. In total, we identified 384 overlapping SNPs covering 224 genes and 7 overlapping InDels covering 7 genes (Figure 5). Among these overlapping regions, we further identified 49 carrier-specific variants (Additional file 13). These variants encompassed 40 genes, including 6 variants covering 2 interferon-stimulated GTPase genes (1 variant within *LOC101903126*, 5 variants within *LOC783920*), 5 variants covering 3 GTPase IMAP family genes (2 variants within *GIMAP8* and *LOC768255*, respectively, and 1 variant within *LOC512867*), 2 variants covering 2 genes related to antigen processing and presentation (*CTSL*, *BLA-DQB*), 2 variants covering 2 genes related to natural killer (NK) cell activity (*NKTR*, *KIR2DS1*), 4 variants covering 4 olfactory receptor family genes (*LOC783328*, *LOC104968693*, *LOC788663*, *OR2W1*), 2 variants covering 2 genes related to chromatin structure and histone methylation (*RBBP5*, *HIST1H2AC_1*), 2 variants covering 2 transcription factor genes (*ZBTB47*, *ZNF205*), 12 variants covering 12 genes related to cell proliferation, differentiation and cytoskeleton remodeling (*CYLC2*, *IRX3*, *DCT*, *LOC100299102*, *TRANK1*, *SPTA1*, *EFCAB13*, *MEGF9*, *EGFL7*, *DCDC2C*, *LOC529969*, *MOS*), and 14 variants covering 11 genes related to diverse signal transduction (1 variant within *IL5RA*, *LOC112443721*, *LOC788915*, *ADAT1*, *NUBPL*, *BPIFA4*, *LOC101906978*, and *SNTG2* repectively; 2 variants within* LOC522174*, *LOC534913*, and *FRRS1L* repectively).

### Cattle–yak haplotype assemblies mapping

To assess potential artefacts arising from hybrid recombination, the clean reads were mapped to cattle–yak (*Bos taurus* × *Bos grunniens*) haplotype 1 and haplotype 2 assemblies after quality filtering. The average genome coverage was greater than 97% (Additional file 14). PCA analysis revealed distinct mutation patterns separating FMDV carriers from noncarriers as well as animal individuals (Additional file 15). We then mapped the identified candidate variants based on the cattle (*Bos taurus*) reference genome (ARS-USD1.2) to two cattle–yak (*Bos taurus* × *Bos grunniens*) haplotype genomes. Among 49 variants identified on the basis of the ARS-USD1.2 genome, 24 variants were consistently present across both cattle–yak haplotype genomes, including 3 variants within 3 olfactory receptor family genes (*LOC783328*, *LOC104968693*, *LOC788663*), 1 variant within 1 gene involved in antigen processing and presentation (*CTSL*), 2 variants covering 2 genes related to NK cell activity (*NKTR*, *KIR2DS1*), 1 variant within 1 gene involved in chromatin structure and histone methylation (*HIST1H2AC_1*), 9 variants covering 9 genes related to cell proliferation, differentiation and cytoskeleton remodeling *(CYLC2*, *IRX3*, *DCT*, *TRANK1*, *SPTA1*, *MEGF9*, *DCDC2C*, *LOC529969*, *EFCAB13*), and 8 variants covering 8 genes related to signal transduction (*IL5RA*, *ADAT1*, *LOC101906978*, *LOC534915*, *BPIFA4*, *FRRS1L*, *NUBPL*, *SNTG2*) (Additional file 16). These results suggest that the prevalence of these 24 variants is independent of hybrid recombination and instead associated solely with the carrier status.

Subsequently, we determined 31 additional differential carrier-specific variants restricted to cattle–yak haplotype genomes (Additional file 17). Notably, 5 additional variants have been identified within 4 olfactory receptor genes (2 variants within *LOC618052*; 1 variant  within *LOC618140*, *LOC100297284*,and *LOC784614* respectively), suggesting that olfactory receptor genes may play a significant role in FMDV colonization in the nasopharynx. The other variants included 4 variants within 4 genes related to regulation of gene transcription and translation (*ZNF74*, *ZNF114*, *ZNF213*, *TCOF1*), 2 variants within 2 genes related to energy metabolism (*MDH1B*, *LOC519145*), 5 variants within 5 genes related to cell development and morphological maintenance (*CEP120*, *DCDC2C*, *IRX6*, *NEK5*, *LAMB3*), 7 variants within 7 genes related to stress resistance (*SEC31A*, *PAQR4*, *LOC100139663*, *LOC404051*, *ENDOV*, *GOLGA5*, *DNAJC4*), 4 variants within 4 genes related to regulation of immune cell function (*LOC508459*, *RGMB*, *INPP5D*, *SIGLEC11*), and 4 variants within 4 genes related to diverse transduction (*LOC101906978*, *TRIM15*, *CACNA2D3*, *LOC517901*). Intriguingly, no single variant was present in all carrier individuals, with multiple distinct mutations coexisting within each individual. This observation suggests that the establishment of persistent FMDV infection is governed by the concerted action of multiple genes rather than being driven by a single genetic factor.

## Discussion

In this study, we conducted whole-genome resequencing on carrier and noncarrier individuals to identify genetic factors associated with the carrier status. Based on the comparative analyses using the cattle (*Bos taurus*) reference genome (ARS-USD1.2) and cattle–yak (*Bos taurus* × *Bos grunniens*) haplotype genomes, we found that significantly differential carrier-specific variants were located in genes encoding olfactory receptors (*LOC783328*, *LOC104968693*, *LOC788663*, *LOC618140, LOC100297284, LOC618052, LOC784614*), as well as genes involved in regulating cell development and morphological maintenance (*CYLC2*, *IRX3*, *DCT*, *TRANK1*, *SPTA1*, *MEGF9*, *DCDC2C*, *LOC529969*, *EFCAB13*, *CEP120*, *IRX6*, *NEK5*, *LAMB3*), transcription and translation regulation (*HIST1H2AC_1*, *ZNF74*, *ZNF114*, *ZNF213*, *TCOF1*), signal transduction (*IL5RA*, *LOC101906978*, *LOC534915*, *BPIFA4*, *FRRS1L*, *SNTG2*, *TRIM15*, *CACNA2D3*, *LOC517901*), energy metabolism (*MDH1B*, *LOC519145*), stress resistance (*ADAT1*, *NUBPL*, *SEC31A*, *PAQR4*, *LOC100139663*, *LOC404051*, *ENDOV*, *GOLGA5*, *DNAJC4*), and immune cell function (*LOC508459*, *RGMB*, *INPP5D*, *SIGLEC11*, *CTSL*, *NKTR*, *KIR2DS1*). These genetic variants potentially determine the innate immune regulatory capacity and the magnitude of adaptive immune activation, thereby conferring distinct antiviral capacities.

Olfactory receptors (ORs) belong to the class A G protein-coupled receptors and constitute the most important chemosensory receptor family responsible for the sense of smell in the nasal olfactory epithelium. The bovine nasopharyngeal mucosal epithelium serves as a critical viral sanctuary for both primary and persistent FMDV infection [7], indicating a potential link between ORs and FMDV infection. Nasal ORs, which are expressed in olfactory sensory neurons, are critical for olfactory cognition and associated neuronal plasticity. The olfactory route has been shown to facilitate the entry of neurotropic viruses, such as poliovirus, herpesviruses, Japanese encephalitis virus, and influenza A virus [52], into the central nervous system. In cattle, FMDV is mainly transmitted through the respiratory tract. Consequently, mutations in OR genes may facilitate viral colonization of the nasopharynx and contribute to viral persistence in carriers.

Transcriptional regulation of genes governs diverse antiviral responses. *HIST1H2AC_1* encodes replication-dependent histones, which are highly expressed just before the S-phase and are then repressed after DNA replication [53]. Mutations in *HIST1H2AC_1* have contributed to carcinogenesis [54]. ZNF74, ZNF114, and ZNF21 belong to an evolutionarily conserved but poorly explored Krueppel C2H2-type ZNF family, and their transcriptional repression by their KRAB domain is critical for early embryonic development [55]. *ZNF74* is a developmentally expressed and commonly deleted gene in DiGeorge syndrome characterized by thymic hypoplasia/aplasia and hypoparathyroidism [56]. ZNF114 interacts with TRIM28 to control self-renewal of human pluripotent stem cells through epigenetic repression of prodifferentiation genes [57]. ZNF213 has been demonstrated to negatively control breast cancer progression [58]. In addition, signal transduction is also crucial for regulating gene expression. Our comprehensive analysis revealed 9 genes (*IL5RA*, *LOC101906978*, *LOC534915*, *BPIFA4*, *FRRS1L*, *SNTG2*, *TRIM15*, *CACNA2D3*, *LOC517901*) that are functionally associated with these processes. For example, *CACNA2D3* encodes a subunit of voltage‐dependent calcium channels and is crucial in calcium signaling, impacting immune cell functions and inflammatory responses. *CACNA2D3* variants have been shown to correlate with asthma and atopic dermatitis multimorbidity in children [59]. *TRIM15* is a TNF-α-induced late response gene and inhibits the TNF-α-induced NF-κB pathway, thereby acting as a feedback modulator to keep the proinflammatory NF-κB pathway under control [60]. Knockdown of *TRIM15* limited virus/RIG-I ligand-induced interferon production and enhanced vesicular stomatitis virus replication [61]. Consequently, genetic variations in these genes may contribute to individual differences in animal development and defense responses to viral infection, influencing the outcomes of viral infections.

FMDV is a prototypical cytolytic virus. Upon infection, FMDV rapidly hijacks the host biosynthetic machinery to facilitate its replication and induces host cell lysis to enable subsequent rounds of infection. Studies have demonstrated that FMDV infection triggers robust cellular stress responses, including cytoskeletal reorganization [62], cell cycle arrest [63], metabolic disturbance [64], endoplasmic reticulum stress [65, 66], autophagy [66, 67], and apoptosis [68]. These processes contribute to the suppression of viral spread and the activation of antiviral immune defenses aimed at viral clearance. Our study identified a catalog of variants in the carrier individuals, which is located in genes involved in regulating cell development and morphological maintenance (*CYLC2*, *IRX3*, *DCT*, *TRANK1*, *SPTA1*, *MEGF9*, *DCDC2C*, *LOC529969*, *EFCAB13*, *CEP120*, *IRX6*, *NEK5*, *LAMB3*), energy metabolism (*MDH1B*, *LOC519145*), and stress resistance (*ADAT1*, *NUBPL*, *SEC31A*, *PAQR4*, *LOC100139663*, *LOC404051*, *ENDOV*, *GOLGA5*, *DNAJC4*). While these genes have not been documented in FMDV-related research, accumulating evidence indicates that their proper function plays a pivotal role in controlling pathogen infections. For instances, variants in *DCDC2C* were associated with susceptibility to salmonella in pig [69]; *SPTA1* mutations were significantly associated with hepatitis D viral infection [70]. NEK5 interacts with mitochondrial proteins and interferes negatively in mitochondrial mediated cell death [71]; in Chlamydia trachomatis infection, GOLGA5 plays a critical role in maintaining the structural integrity of the Golgi apparatus [72]. Therefore, genetic mutations in key components of these biological processes potentially compromise host's intrinsic defense mechanisms, leading to FMDV persistence.

In general, innate immunity primarily functions by secreting cytokines to recruit and activate immune cells, and adaptive immunity is responsible for the ultimate clearance of viral infections. An optimal immune response is characterized by efficient antigen presentation as well as strong and well-balanced humoral and cellular immunity. Here, we identified seven genetic variants in genes involved in regulating antigen presentation (*CTSL*),  NK (*NKTR*, *KIR2DS1*) and T (*LOC508459*, *RGMB*, *INPP5D*, *SIGLEC11*) cell function. Cathepsin L (CTSL) is involved in MHC II-dependent immune responses by cleaving the chaperone molecule invariant chain, which supports MHC II assembly in the endoplasmic reticulum into intermediate products [73]. Accordingly, functional mutations in *CSTL* affect FMDV antigen presentation and immune cell activation. NK cells play critical roles in innate antiviral immunity by recognizing and lysing virus-infected cells. A lack of or deficiency in NK cell function has been demonstrated to lead to increased susceptibility to viral infection [74]. NKTR and KIR2DS1 are important receptors that regulate NK cell cytotoxicity. Genetic variants in *KIR2DS1* are associated with increased susceptibility to HCV infection in a high-risk Chinese population [75]. In contrast to the suppressed NK activity observed in FMDV-infected swine, NK cells play a role in host immune responses against FMDV in cattle [76]. Moreover, studies have shown that the effector functions of *INPP5D*-deficient NK and T cells are compromised in vivo [77]. Collectively, the variations in genes involved in regulating immune cell function potentially impair antiviral cellular immune activation, thereby facilitating viral persistence.

While our screened genes did not overlap with those previously reported in transcriptomic datasets from tissues of FMDV persistently infected individuals [35, 36, 78], they are all involved in comparable biological processes that are intimately linked to the host's antiviral defenses and self-protection mechanisms. Mutations in these genes may impair the host's antiviral response and self-repair capacity, thereby facilitating long-term viral persistence. Another possible explanation is that genes showing marked changes in transcriptomic levels are generally downstream effector genes, whereas most of the variants we identified are located in key genes that regulate these downstream effectors.

In conclusion, our study comprehensively evaluated genetic variants associated with the carrier status through WGS analyses. These findings provide novel insights into the genetic architecture underlying persistence and offer avenues for improved prevention and control strategies. Although we investigated susceptibility to persistent FMDV infection using high-throughput WGS, we were aware of the limitations of this study, such as the small sample size and the absence of interspecies and intersex comparisons. Constraints of laboratory capacity and eradication policies make it difficult to obtain large numbers of samples from animals with established persistent infections.

## Supplementary Information

**Additional file 1**. **Basic information of enrolled subjects.**

**Additional file 2**. **Summary of whole genome sequences mapped to the cattle reference genome.**

**Additional file 3**. **Distribution of SNPs identified in cattle individuals within various genomic regions based on the cattle reference genome.**

**Additional file 4**. **Distribution of Indels identified in cattle individuals within various genomic regions based on the cattle reference genome.**

**Additional file 5**. **Population genetic structure**. **A** **Principal component analysisof the distribution of all genotyped SNPs from all resequencing samples.** **B** **Kinship of all resequencing samples. CR, carriers; NCR, noncarriers.**

**Additional file 6**. **Kinship analysis of all subjected animals based on the cattle reference genome.**

**Additional file 7**. **Genome-wide association study.** **A** **Quantile–quantile plot of GWAS using GLM, MLM, and FarmCPU tests.** **B Manhattan plots showing SNPs significantly associated with the carrier state. The X-axis represents the chromosome, and the Y-axis indicates −log**_**10**_. **Dots over the dotted line indicate significantly associated SNPs.**

**Additional file 8. Variants significantly associated with the FMDV carrier state identified by a GLM model based on the cattle reference genome.**

**Additional file 9**. **Differential missense SNPs with** ***p*-value < 0.01 of Fisher’s exact test between carriers and noncarriers based on the cattle reference genome.**

**Additional file 10**. **Differential missense Indels with *p*-value < 0.01 of Fisher’s exact test between carriers and noncarriers based on the cattle reference genome.**

**Additional file 11**. **SNPs with the top 1% *F***_**ST**_ **values represent extreme allele frequency differences between carriers and noncarriers based on the cattle reference genome.**

**Additional file 12**. **Indels with the top 1% *F***_**ST**_ **values represent extreme allele frequency differences between carriers and noncarriers based on the cattle reference genome.**

**Additional file 13**. **Genetic variations exclusively identified in carrier individuals based on the cattle reference genome.**

**Additional file 14**. **Summary of whole genome sequences mapped to cattle–yak haplotype genomes.**

**Additional file 15**. **Principal component analysis of the SNP distribution from all resequencing samples.A** **The PCA representation of haplotype genomes 1 and 2 separately.** **B** **The overlay representation of PCA based on haplotype genomes 1 and 2.**

**Additional file 16**. **Genetic variations extensively identified in carrier individuals based on the cattle reference genome and cattle–yak haplotype genomes.**

**Additional file 17**.** Genetic variations exclusively identified in carrier individuals based on cattle–yak haplotype genomes.**

## Data Availability

The raw WGS sequencing data have been deposited in the Genome Sequence Archive of the National Genomics Data Center [79] under accession number CRA020792. All other data are present in the paper and/or the Supplementary Information. All analyses in this manuscript were conducted by open-source softwares.

## References

[1] Alexandersen S, Mowat N (2005) Foot-and-mouth disease: host range and pathogenesis. Curr Top Microbiol Immunol 288:9–42 15648173 10.1007/3-540-27109-0_2

[2] Knight-Jones TJD, Rushton J (2013) The economic impacts of foot and mouth disease—What are they, how big are they and where do they occur? Prev Vet Med 112:161–173 23958457 10.1016/j.prevetmed.2013.07.013 PMC3989032

[3] Knight-Jones TJD, McLaws M, Rushton J (2017) Foot-and-mouth disease impact on smallholders—What do we know, what don’t we know and how can we find out more? Transbound Emerg Dis 64:1079–1094 27167976 10.1111/tbed.12507 PMC5516236

[4] Stenfeldt C, Arzt J (2020) The carrier conundrum; a review of recent advances and persistent gaps regarding the carrier state of foot-and-mouth disease virus. Pathogens 9:167 32121072 10.3390/pathogens9030167 PMC7157498

[5] Arzt J, Juleff N, Zhang Z, Rodriguez LL (2011) The pathogenesis of foot-and-mouth disease I: viral pathways in cattle. Transbound Emerg Dis 58:291–304 21366894 10.1111/j.1865-1682.2011.01204.x

[6] WOAH (2022) Terrestrial manual: foot and mouth disease

[7] Stenfeldt C, Hartwig EJ, Smoliga GR, Palinski R, Silva EB, Bertram MR, Fish IH, Pauszek SJ, Arzt J (2018) Contact challenge of cattle with foot-and-mouth disease virus validates the role of the nasopharyngeal epithelium as the site of primary and persistent infection. mSphere 3:e00493-18 30541776 10.1128/mSphere.00493-18 PMC6291620

[8] Stenfeldt C, Eschbaumer M, Rekant SI, Pacheco JM, Smoliga GR, Hartwig EJ, Rodriguez LL, Arzt J (2016) The foot-and-mouth disease carrier state divergence in cattle. J Virol 90:6344–6364 27147736 10.1128/JVI.00388-16 PMC4936139

[9] Eschbaumer M, Stenfeldt C, Rekant SI, Pacheco JM, Hartwig EJ, Smoliga GR, Kenney MA, Golde WT, Rodriguez LL, Arzt J (2016) Systemic immune response and virus persistence after foot-and-mouth disease virus infection of naïve cattle and cattle vaccinated with a homologous adenovirus-vectored vaccine. BMC Vet Res 12:205 27634113 10.1186/s12917-016-0838-x PMC5025598

[10] Moonen P, Schrijver R (2000) Carriers of foot-and-mouth disease virus: a review. Vet Q 22:193–197 11087128 10.1080/01652176.2000.9695056

[11] Stenfeldt C, Pacheco JM, Singanallur NB, Vosloo W, Rodriguez LL, Arzt J (2019) Virulence beneath the fleece; a tale of foot-and-mouth disease virus pathogenesis in sheep. PLoS One 14:e0227061 31891626 10.1371/journal.pone.0227061 PMC6938329

[12] Maree F, de Klerk-Lorist L-M, Gubbins S, Zhang F, Seago J, Pérez-Martín E, Reid L, Scott K, van Schalkwyk L, Bengis R, Charleston B, Juleff N (2016) Differential persistence of foot-and-mouth disease virus in African buffalo is related to virus virulence. J Virol 90:5132–5140 26962214 10.1128/JVI.00166-16 PMC4859713

[13] Arzt J, Belsham GJ, Lohse L, Bøtner A, Stenfeldt C (2018) Transmission of foot-and-mouth disease from persistently infected carrier cattle to naive cattle via transfer of oropharyngeal fluid. mSphere 3:e00365-18 30209130 10.1128/mSphere.00365-18 PMC6135961

[14] Jolles A, Gorsich E, Gubbins S, Beechler B, Buss P, Juleff N, de Klerk-Lorist L-M, Maree F, Perez-Martin E, van Schalkwyk OL, Scott K, Zhang F, Medlock J, Charleston B (2021) Endemic persistence of a highly contagious pathogen: foot-and-mouth disease in its wildlife host. Science 374:104–109 34591637 10.1126/science.abd2475

[15] Dawe PS, Sorensen K, Ferris NP, Barnett IT, Armstrong RM, Knowles NJ (1994) Experimental transmission of foot-and-mouth disease virus from carrier African buffalo (*Syncerus caffer*) to cattle in Zimbabwe. Vet Rec 134:211–215 8171808 10.1136/vr.134.9.211

[16] Dawe PS, Flanagan FO, Madekurozwa RL, Sorensen KJ, Anderson EC, Foggin CM, Ferris NP, Knowles NJ (1994) Natural transmission of foot-and-mouth disease virus from African buffalo (*Syncerus caffer*) to cattle in a wildlife area of Zimbabwe. Vet Rec 134:230–232 8197679 10.1136/vr.134.10.230

[17] Tenzin DA, Vernooij H, Bouma A, Stegeman A (2008) Rate of foot-and-mouth disease virus transmission by carriers quantified from experimental data. Risk Anal 28:303–309 18419650 10.1111/j.1539-6924.2008.01020.x

[18] Arzt J, Fish IH, Bertram MR, Smoliga GR, Hartwig EJ, Pauszek SJ, Holinka-Patterson L, Diaz-San Segundo FC, Sitt T, Rieder E, Stenfeldt C (2021) Simultaneous and staggered foot-and-mouth disease virus coinfection of cattle. J Virol 95:e0165021 34586864 10.1128/JVI.01650-21 PMC8610595

[19] Stenfeldt C, Fish IH, Rodriguez-Calzada M, Medina G, Richt JA, Arzt J (2025) Differential mosaicism of recombinant foot-and-mouth disease viruses resulting from heterologous superinfection of cattle. J Virol 99:e0221324 39932317 10.1128/jvi.02213-24 PMC11915824

[20] Cortey M, Ferretti L, Pérez-Martín E, Zhang F, de Klerk-Lorist L-M, Scott K, Freimanis G, Seago J, Ribeca P, van Schalkwyk L, Juleff ND, Maree FF, Charleston B (2019) Persistent infection of African buffalo (*Syncerus caffer*) with foot-and-mouth disease virus: limited viral evolution and no evidence of antibody neutralization escape. J Virol 93:e00563-19 31092573 10.1128/JVI.00563-19 PMC6639274

[21] Arzt J, Fish I, Pauszek SJ, Johnson SL, Chain PS, Rai DK, Rieder E, Goldberg TL, Rodriguez LL, Stenfeldt C (2019) The evolution of a super-swarm of foot-and-mouth disease virus in cattle. PLoS One 14:e0210847 31022193 10.1371/journal.pone.0210847 PMC6483180

[22] Ramirez-Carvajal L, Pauszek SJ, Ahmed Z, Farooq U, Naeem K, Shabman RS, Stockwell TB, Rodriguez LL (2018) Genetic stability of foot-and-mouth disease virus during long-term infections in natural hosts. PLoS One 13:e0190977 29390015 10.1371/journal.pone.0190977 PMC5794060

[23] Parthiban AR, Mahapatra M, Parida S (2015) Complete genome sequences of serotype O foot-and-mouth disease viruses recovered from experimental persistently infected cattle. Genome Announc 3:e00606-15 26159521 10.1128/genomeA.00606-15 PMC4498108

[24] Pauszek SJ, Eschbaumer M, Brito BP, Ferreira H, Vu LT, Phuong NTT, Hoang BH, Tho ND, Dong PV, Minh PQ, Long NT, Dung DH, Rodriguez LL, Arzt J (2016) Site-specific substitution (Q172R) in the VP1 protein of FMDV isolates collected from asymptomatic carrier ruminants in Vietnam. Virol Rep 6:90–96

[25] Horsington J, Zhang Z (2007) Consistent change in the B-C loop of VP2 observed in foot-and-mouth disease virus from persistently infected cattle: implications for association with persistence. Virus Res 125:114–118 17241682 10.1016/j.virusres.2006.12.008

[26] Litz B, Forth LF, Pfaff F, Beer M, Eschbaumer M (2025) Distinct mutations emerge in the genome of serotype O foot-and-mouth disease virus during persistence in cattle. J Virol 99:e0142224 39918330 10.1128/jvi.01422-24 PMC11915810

[27] Litz B, Sehl-Ewert J, Breithaupt A, Landmesser A, Pfaff F, Romey A, Blaise-Boisseau S, Beer M, Eschbaumer M (2024) Leaderless foot-and-mouth disease virus serotype O did not cause clinical disease and failed to establish a persistent infection in cattle. Emerg Microbes Infect 13:2348526 38683015 10.1080/22221751.2024.2348526 PMC11100440

[28] Fish I, Stenfeldt C, Spinard E, Medina GN, Azzinaro PA, Bertram MR, Holinka L, Smoliga GR, Hartwig EJ, de Los Santos T, Arzt J (2022) Foot-and-mouth disease virus interserotypic recombination in superinfected carrier cattle. Pathogens 11:644 35745498 10.3390/pathogens11060644 PMC9231328

[29] Stenfeldt C, Heegaard PMH, Stockmarr A, Tjørnehøj K, Belsham GJ (2011) Analysis of the acute phase responses of serum amyloid a, haptoglobin and type 1 interferon in cattle experimentally infected with foot-and-mouth disease virus serotype O. Vet Res 42:66 21592356 10.1186/1297-9716-42-66 PMC3123197

[30] Windsor MA, Carr BV, Bankowski B, Gibson D, Reid E, Hamblin P, Gubbins S, Juleff N, Charleston B (2011) Cattle remain immunocompetent during the acute phase of foot-and-mouth disease virus infection. Vet Res 42:108 22014145 10.1186/1297-9716-42-108 PMC3207891

[31] Arzt J, Pacheco JM, Smoliga GR, Tucker MT, Bishop E, Pauszek SJ, Hartwig EJ, de los Santos T, Rodriguez LL (2014) Foot-and-mouth disease virus virulence in cattle is co-determined by viral replication dynamics and route of infection. Virology 452–453:12–22 24606678 10.1016/j.virol.2014.01.001

[32] Biswal JK, Nardo AD, Taylor G, Paton DJ, Parida S (2021) Development and validation of a mucosal antibody (IgA) test to identify persistent infection with foot-and-mouth disease virus. Viruses 13:814 34062811 10.3390/v13050814 PMC8147266

[33] Maddur MS, Gajendragad MR, Kishore S, Chockalingam AK, Suryanarayana VVS, Gopalakrishna S, Singh N (2008) Enhanced mucosal immune response in cattle persistently infected with foot-and-mouth disease virus. Vet Immunol Immunopathol 125:337–343 18656268 10.1016/j.vetimm.2008.05.031

[34] Parida S, Anderson J, Cox SJ, Barnett PV, Paton DJ (2006) Secretory IgA as an indicator of oro-pharyngeal foot-and-mouth disease virus replication and as a tool for post vaccination surveillance. Vaccine 24:1107–1116 16203061 10.1016/j.vaccine.2005.09.006

[35] Stenfeldt C, Eschbaumer M, Smoliga GR, Rodriguez LL, Zhu J, Arzt J (2017) Clearance of a persistent picornavirus infection is associated with enhanced pro-apoptotic and cellular immune responses. Sci Rep 7:17800 29259271 10.1038/s41598-017-18112-4 PMC5736604

[36] Eschbaumer M, Stenfeldt C, Smoliga GR, Pacheco JM, Rodriguez LL, Li RW, Zhu J, Arzt J (2016) Transcriptomic analysis of persistent infection with foot-and-mouth disease virus in cattle suggests impairment of apoptosis and cell-mediated immunity in the nasopharynx. PLoS One 11:e0162750 27643611 10.1371/journal.pone.0162750 PMC5028045

[37] Maddur MS, Kishore S, Chockalingam AK, Gopalakrishna S, Singh N, Suryanarayana VVS, Gajendragad MR (2010) The relationship between cellular immune response to foot-and-mouth disease virus Asia 1 and viral persistence in Indian cattle (*Bosindicus*). Res Vet Sci 89:36–40 20189208 10.1016/j.rvsc.2010.01.018

[38] Cai Y, Chen Q, Zhou W, Chu C, Ji W, Ding Y, Xu J, Ji Z, You H, Wang J (2014) Association analysis of polymorphisms in OAS1 with susceptibility and severity of hand, foot and mouth disease. Int J Immunogenet 41:384–392 25059424 10.1111/iji.12134

[39] Zou R, Zhang G, Li S, Wang W, Yuan J, Li J, Wang Y, Lin Y, Deng Y, Zhou B, Gao GF, Liu Y (2015) A functional polymorphism in IFNAR1 gene is associated with susceptibility and severity of HFMD with EV71 infection. Sci Rep 5:18541 26679744 10.1038/srep18541 PMC4683517

[40] Zhang X, Xu H, Chen X, Li X, Wang X, Ding S, Zhang R, Liu L, He C, Zhuang L, Li H, Zhang P, Yang H, Li T, Liu W, Cao W (2014) Association of functional polymorphisms in the MxA gene with susceptibility to enterovirus 71 infection. Hum Genet 133:187–197 24085612 10.1007/s00439-013-1367-3 PMC7088390

[41] Song J, Liu Y, Guo Y, Liu P, Li F, Yang C, Pan X, Yi L, Fan F, Zhao H, Chen Z (2022) Association of the IRAK4 rs4251545 genetic polymorphism with severity of enterovirus-71 infection in Chinese children. Immun Inflamm Dis 10:e614 35478439 10.1002/iid3.614 PMC9017637

[42] Yen T-Y, Shih W-L, Huang Y-C, Lee J-T, Huang L-M, Chang L-Y (2018) Polymorphisms in enterovirus 71 receptors associated with susceptibility and clinical severity. PLoS One 13:e0206769 30395634 10.1371/journal.pone.0206769 PMC6218064

[43] Asgari S, Schlapbach LJ, Anchisi S, Hammer C, Bartha I, Junier T, Mottet-Osman G, Posfay-Barbe KM, Longchamp D, Stocker M, Cordey S, Kaiser L, Riedel T, Kenna T, Long D, Schibler A, Telenti A, Tapparel C, McLaren PJ, Garcin D, Fellay J (2017) Severe viral respiratory infections in children with IFIH1 loss-of-function mutations. Proc Natl Acad Sci USA 114:8342–8347 28716935 10.1073/pnas.1704259114 PMC5547624

[44] Lei W, Liang Q, Jing L, Wang C, Wu X, He H (2012) BoLA-DRB3 gene polymorphism and FMD resistance or susceptibility in Wanbei cattle. Mol Biol Rep 39:9203–9209 22744423 10.1007/s11033-012-1793-7 PMC3404275

[45] Chaudhary Y, Khuntia P, Kaul R (2022) Susceptibility to foot and mouth disease virus infection in vaccinated cattle, and host BoLA A and BoLA DRB3 genes polymorphism. Virusdisease 33:65–75 35493756 10.1007/s13337-021-00754-8 PMC9005608

[46] Singh R, Alex R, Singh U, Kumar S, Sengar GS, Raja TV, Alyethodi RR, Kumar A, Deb R (2018) A synonymous mutation at bovine alpha vitronectin domain of integrin host receptor (ITGAV) gene effect the susceptibility of foot-and-mouth disease in crossbred cattle. Adv Exp Med Biol 1057:41–45 28567614 10.1007/5584_2017_47

[47] Singh R, Deb R, Singh U, Raja TV, Alex R, Kumar S, Chakraborti S, Alyethodi RR, Sharma S, Sengar G (2015) Heterozygosity at the SNP (rs136500299) of ITGB6 receptor gene possibly influences the susceptibility among crossbred bull to foot and mouth disease infection. Virusdisease 26:48–54 26436121 10.1007/s13337-015-0249-9 PMC4585059

[48] Othman OE, Khodary MG, El-Deeb AH, Hussein HA (2018) Five BoLA-DRB3 genotypes detected in Egyptian buffalo infected with foot and mouth disease virus serotype O. J Genet Eng Biotechnol 16:513–518 30733768 10.1016/j.jgeb.2018.02.009 PMC6353717

[49] Lee B-Y, Lee K-N, Lee T, Park J-H, Kim S-M, Lee H-S, Chung D-S, Shim H-S, Lee H-K, Kim H (2015) Bovine genome-wide association study for genetic elements to resist the infection of foot-and-mouth disease in the field. Asian-Australas J Anim Sci 28:166–170 25557811 10.5713/ajas.14.0383 PMC4283160

[50] Saravanan S, Guleria N, Ranjitha HB, Sreenivasa BP, Hosamani M, Prieto C, Umapathi V, Santosh HK, Behera S, Dhanesh VV, Krishna GS, Gopinath S, Kolte A, Bayry J, Sanyal A, Basagoudanavar SH (2021) Induction of antiviral and cell mediated immune responses significantly reduce viral load in an acute foot-and-mouth disease virus infection in cattle. Genomics 113:4254–4266 34757126 10.1016/j.ygeno.2021.10.016

[51] Danecek P, Auton A, Abecasis G, Albers CA, Banks E, DePristo MA, Handsaker RE, Lunter G, Marth GT, Sherry ST, McVean G, Durbin R (2011) The variant call format and VCFtools. Bioinformatics 27:2156–2158 21653522 10.1093/bioinformatics/btr330 PMC3137218

[52] Durrant DM, Ghosh S, Klein RS (2016) The olfactory bulb: an immunosensory effector organ during neurotropic viral infections. ACS Chem Neurosci 7:464–469 27058872 10.1021/acschemneuro.6b00043 PMC5775964

[53] Wu RS, Bonner WM (1981) Separation of basal histone synthesis from S-phase histone synthesis in dividing cells. Cell 27:321–330 7199388 10.1016/0092-8674(81)90415-3

[54] Singh R, Mortazavi A, Telu KH, Nagarajan P, Lucas DM, Thomas-Ahner JM, Clinton SK, Byrd JC, Freitas MA, Parthun MR (2013) Increasing the complexity of chromatin: functionally distinct roles for replication-dependent histone H2A isoforms in cell proliferation and carcinogenesis. Nucleic Acids Res 41:9284–9295 23956221 10.1093/nar/gkt736 PMC3814372

[55] Iuchi S (2001) Three classes of C2H2 zinc finger proteins. Cell Mol Life Sci 58:625–635 11361095 10.1007/PL00000885 PMC11146492

[56] Grondin B, Bazinet M, Aubry M (1996) The KRAB zinc finger gene ZNF74 encodes an RNA-binding protein tightly associated with the nuclear matrix. J Biol Chem 271:15458–15467 8663113 10.1074/jbc.271.26.15458

[57] Oleksiewicz U, Gładych M, Raman AT, Heyn H, Mereu E, Chlebanowska P, Andrzejewska A, Sozańska B, Samant N, Fąk K, Auguścik P, Kosiński M, Wróblewska JP, Tomczak K, Kulcenty K, Płoski R, Biecek P, Esteller M, Shah PK, Rai K, Wiznerowicz M (2017) TRIM28 and interacting KRAB-ZNFs control self-renewal of human pluripotent stem cells through epigenetic repression of pro-differentiation genes. Stem Cell Reports 9:2065–2080 29198826 10.1016/j.stemcr.2017.10.031 PMC5785758

[58] Liu Y, Su P, Zhao W, Li X, Yang X, Fan J, Yang H, Yan C, Mao L, Ding Y, Zhu J, Niu Z, Zhuang T (2021) ZNF213 negatively controls triple negative breast cancer progression via Hippo/YAP signaling. Cancer Sci 112:2714–2727 33939216 10.1111/cas.14916 PMC8253295

[59] Kim DY, Lee S, Jung JH, Sub Y, Lee S, Kim EG, Kim MN, Kim SY, Kim YH, Sohn MH, Gee HY, Kim KW (2025) GWAS identifies CACNA2D3 associated with asthma and atopic dermatitis multimorbidity in children. Allergy 80:1776–1781 39868909 10.1111/all.16483 PMC12186590

[60] Roy M, Singh K, Shinde A, Singh J, Mane M, Bedekar S, Tailor Y, Gohel D, Vasiyani H, Currim F, Singh R (2021) TNF-α-induced E3 ligase, TRIM15 inhibits TNF-α-regulated NF-κB pathway by promoting turnover of K63 linked ubiquitination of TAK1. Cell Signal 91:110210 34871740 10.1016/j.cellsig.2021.110210

[61] Uchil PD, Hinz A, Siegel S, Coenen-Stass A, Pertel T, Luban J, Mothes W (2012) TRIM protein-mediated regulation of inflammatory and innate immune signaling and its association with antiretroviral activity. J Virol 87:257–272 23077300 10.1128/JVI.01804-12 PMC3536418

[62] Armer H, Moffat K, Wileman T, Belsham GJ, Jackson T, Duprex WP, Ryan M, Monaghan P (2008) Foot-and-mouth disease virus, but not bovine enterovirus, targets the host cell cytoskeleton via the nonstructural protein 3Cpro. J Virol 82:10556–10566 18753210 10.1128/JVI.00907-08 PMC2573224

[63] Xin X, Wang H, Han L, Wang M, Fang H, Hao Y, Li J, Zhang H, Zheng C, Shen C (2018) Single-cell analysis of the impact of host cell heterogeneity on infection with foot-and-mouth disease virus. J Virol 92:e00179-18 29444939 10.1128/JVI.00179-18 PMC5899210

[64] Lv B, Yuan Y, Yang Z, Wang X, Hu J, Sun Y, Du H, Liu X, Duan H, Ding R, Pan Z, Tang X-F, Shen C (2024) Stearoyl coenzyme A desaturase 1 (SCD1) regulates foot-and-mouth disease virus replication by modulating host cell lipid metabolism and viral protein 2C-mediated replication complex formation. J Virol 98:e0090224 39324793 10.1128/jvi.00902-24 PMC11495015

[65] Han S, Mao L, Liao Y, Sun S, Zhang Z, Mo Y, Liu H, Zhi X, Lin S, Seo HS, Guo H (2019) Sec62 suppresses foot-and-mouth disease virus proliferation by promotion of IRE1α-RIG-I antiviral signaling. J Immunol 203:429–440 31167774 10.4049/jimmunol.1801546

[66] Wu Je, Zhang Z, Teng Z, Abdullah SW, Sun S, Guo H (2021) Sec62 regulates endoplasmic reticulum stress and autophagy balance to affect foot-and-mouth disease virus replication. Front Cell Infect Microbiol 11:707107 34532300 10.3389/fcimb.2021.707107 PMC8438241

[67] Fan X, Han S, Yan D, Gao Y, Wei Y, Liu X, Liao Y, Guo H, Sun S (2017) Foot-and-mouth disease virus infection suppresses autophagy and NF-кB antiviral responses via degradation of ATG5-ATG12 by 3Cpro. Cell Death Dis 8:e2561 10.1038/cddis.2016.489 PMC5386389 28102839

[68] Ren X, Yin M, Zhao Q, Zheng Z, Wang H, Lu Z, Li X, Qian P (2023) Foot-and-mouth disease virus induces porcine gasdermin E-mediated pyroptosis through the protease activity of 3Cpro. J Virol 97:e0068623 37367489 10.1128/jvi.00686-23 PMC10373541

[69] Schut CH, Farzan A, Fraser RS, Ainslie-Garcia MH, Friendship RM, Lillie BN (2020) Identification of single-nucleotide variants associated with susceptibility to *Salmonella* in pigs using a genome-wide association approach. BMC Vet Res 16:138 32414370 10.1186/s12917-020-02344-0 PMC7227190

[70] Candia J, Bayarsaikhan E, Tandon M, Budhu A, Forgues M, Tovuu L-O, Tudev U, Lack J, Chao A, Chinburen J, Wang XW (2020) The genomic landscape of Mongolian hepatocellular carcinoma. Nat Commun 11:4383 32873799 10.1038/s41467-020-18186-1 PMC7462863

[71] Melo Hanchuk TD, Papa PF, La Guardia PG, Vercesi AE, Kobarg J (2015) Nek5 interacts with mitochondrial proteins and interferes negatively in mitochondrial mediated cell death and respiration. Cell Signal 27:1168–1177 25725288 10.1016/j.cellsig.2015.02.021

[72] Zhu H, Li H, Wang P, Chen M, Huang Z, Li K, Li Y, He J, Han J, Zhang Q (2014) Persistent and acute chlamydial infections induce different structural changes in the Golgi apparatus. Int J Med Microbiol 304:577–585 24780199 10.1016/j.ijmm.2014.03.002

[73] Zhao K, Sun Y, Zhong S, Luo J-L (2024) The multifaceted roles of cathepsins in immune and inflammatory responses: implications for cancer therapy, autoimmune diseases, and infectious diseases. Biomark Res 12:165 39736788 10.1186/s40364-024-00711-9 PMC11687005

[74] Bittleman DB, Maves KK, Bertolatus JA, Bonsib SM, Densen P, Ballas ZK, Weiler JM (1994) Recurrent infections, pericarditis and renal disease in a patient with total C2 deficiency and decreased NK cell function consistent with acute rheumatic fever and systemic lupus erythematosus. Ann Rheum Dis 53:280–281 8203960 10.1136/ard.53.4.280 PMC1005310

[75] Shen C, Ge Z, Dong C, Wang C, Shao J, Cai W, Huang P, Fan H, Li J, Zhang Y, Yue M (2021) Genetic variants in KIR/HLA-C genes are associated with the susceptibility to HCV infection in a high-risk Chinese population. Front Immunol 12:632353 34220799 10.3389/fimmu.2021.632353 PMC8253047

[76] Patch JR, Dar PA, Waters R, Toka FN, Barrera J, Schutta C, Kondabattula G, Golde WT (2014) Infection with foot-and-mouth disease virus (FMDV) induces a natural killer (NK) cell response in cattle that is lacking following vaccination. Comp Immunol Microbiol Infect Dis 37:249–257 25150134 10.1016/j.cimid.2014.07.004

[77] Gumbleton M, Sudan R, Fernandes S, Engelman RW, Russo CM, Chisholm JD, Kerr WG (2017) Dual enhancement of T and NK cell function by pulsatile inhibition of SHIP1 improves antitumor immunity and survival. Sci Signal 10:eaam5353 29018171 10.1126/scisignal.aam5353

[78] Zhu JJ, Stenfeldt C, Bishop EA, Canter JA, Eschbaumer M, Rodriguez LL, Arzt J (2020) Mechanisms of maintenance of foot-and-mouth disease virus persistence inferred from genes differentially expressed in nasopharyngeal epithelia of virus carriers and non-carriers. Front Vet Sci 7:340 32637426 10.3389/fvets.2020.00340 PMC7318773

[79] The genome sequence archive in the National Genomics Data Center, China National Center for Bioinformation. https://ngdc.cncb.ac.cn/gsa

