# Supplementary material for: The whole genome sequencing offers insights into the susceptibility to the foot-and-mouth disease virus carrier state
Source: Vet Res. 2026 Jan 3;57:26. doi: 10.1186/s13567-025-01697-4 (PMC12866560; doi:10.1186/s13567-025-01697-4)
Supplement: Supplementary file 5 — Additional file 5. Population genetic structure. A Principal component analysisof the distribution of all genotyped SNPs from all resequencing samples. B Kinship of all resequencing samples. CR, carriers; NCR, noncarriers. [file 13567_2025_1697_MOESM5_ESM.docx]

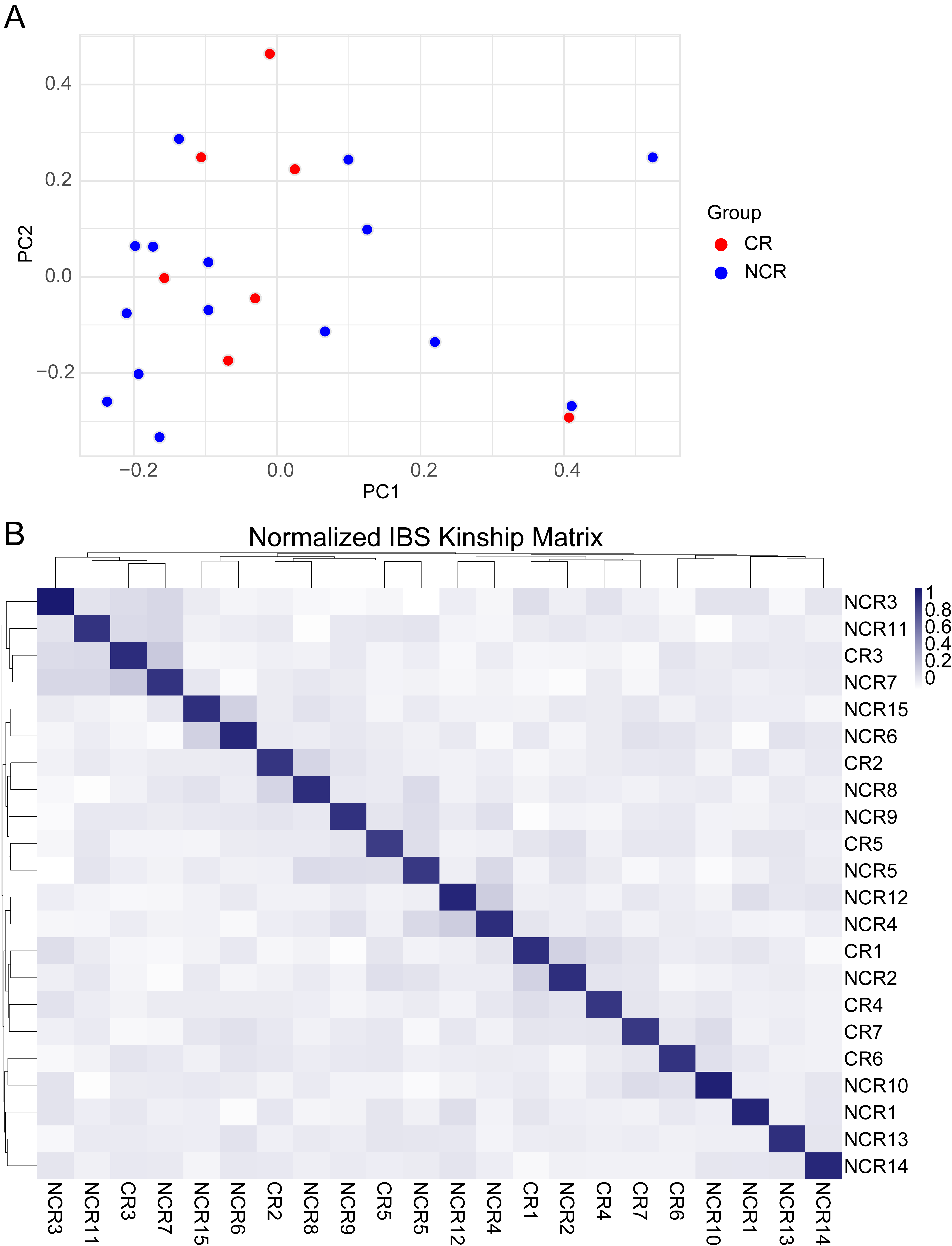


**Additional file 5.** **Population genetic structure.** (A) Principal component analysis (PCA) of the distribution of all genotyped SNPs from all resquenced samples. (B) Kinship of all resquenced samples. CR, carriers; NCR, noncarriers.
