# Supplementary material for: The whole genome sequencing offers insights into the susceptibility to the foot-and-mouth disease virus carrier state
Source: Vet Res. 2026 Jan 3;57:26. doi: 10.1186/s13567-025-01697-4 (PMC12866560; doi:10.1186/s13567-025-01697-4)
Supplement: Supplementary file 7 — Additional file 7. Genome-wide association study. A Quantile–quantile plot of GWAS using GLM, MLM, and FarmCPU tests. B Manhattan plots showing SNPs significantly associated with the carrier state. The X-axis represents the chromosome, and the Y-axis indicates −log10. Dots over the dotted line indicate significantly associated SNPs. [file 13567_2025_1697_MOESM7_ESM.docx]

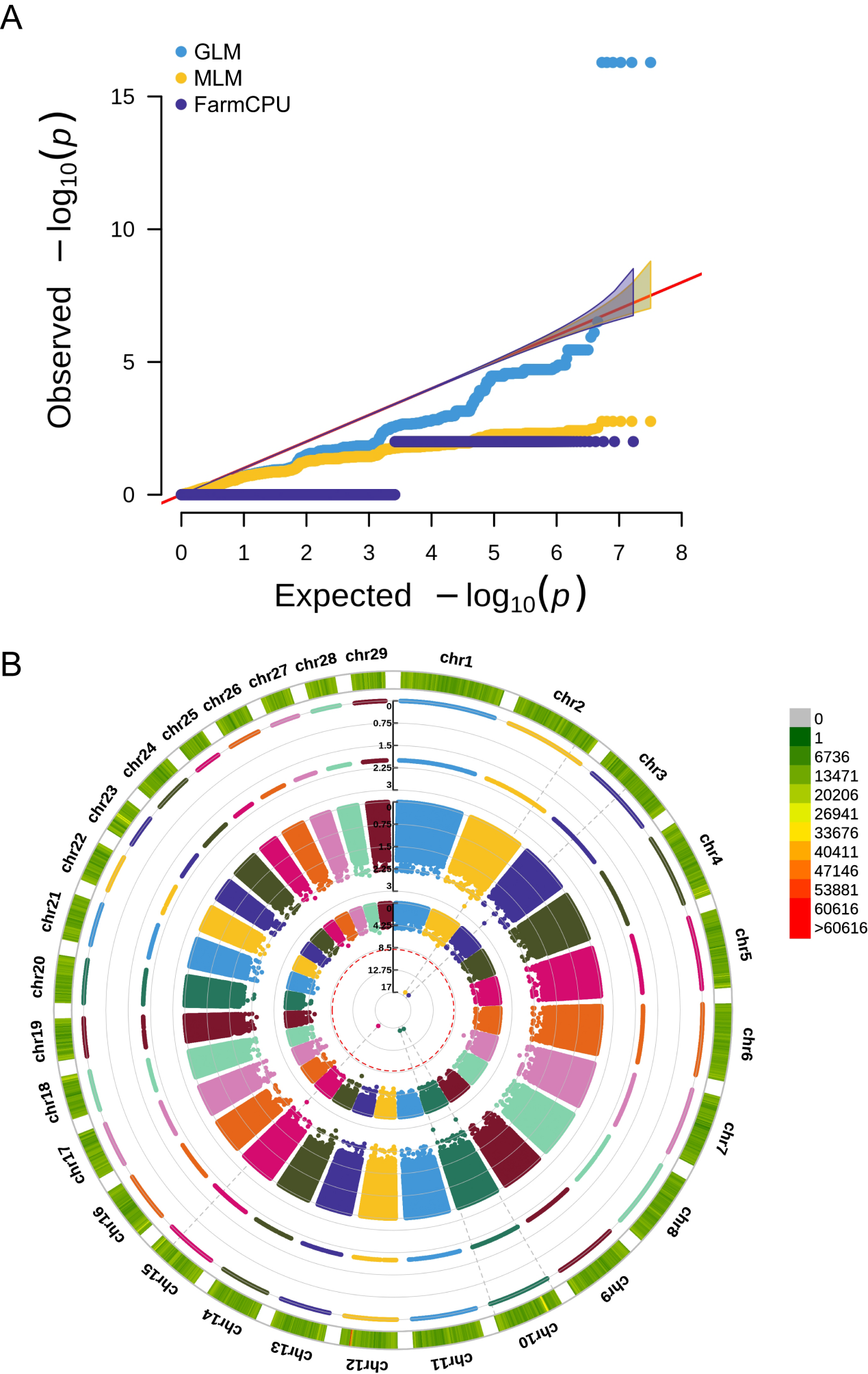


**Additional file 7. Genome-wide association study.** (A) Quantile–quantile plot of GWAS using GLM, MLM and FarmCPU tests. (B) Manhattan plots showing SNPs significantly associated with the carrier state. The X-axis represents chromosome and the Y-axis indicated −log_10_(p value). Dots over the dotted line indicate significantly associated SNPs.
