# Supplementary material for: The whole genome sequencing offers insights into the susceptibility to the foot-and-mouth disease virus carrier state
Source: Vet Res. 2026 Jan 3;57:26. doi: 10.1186/s13567-025-01697-4 (PMC12866560; doi:10.1186/s13567-025-01697-4)
Supplement: Supplementary file 15 — Additional file 15. Principal component analysis of the SNP distribution from all resequencing samples.A The PCA representation of haplotype genomes 1 and 2 separately. B The overlay representation of PCA based on haplotype genomes 1 and 2. [file 13567_2025_1697_MOESM15_ESM.docx]

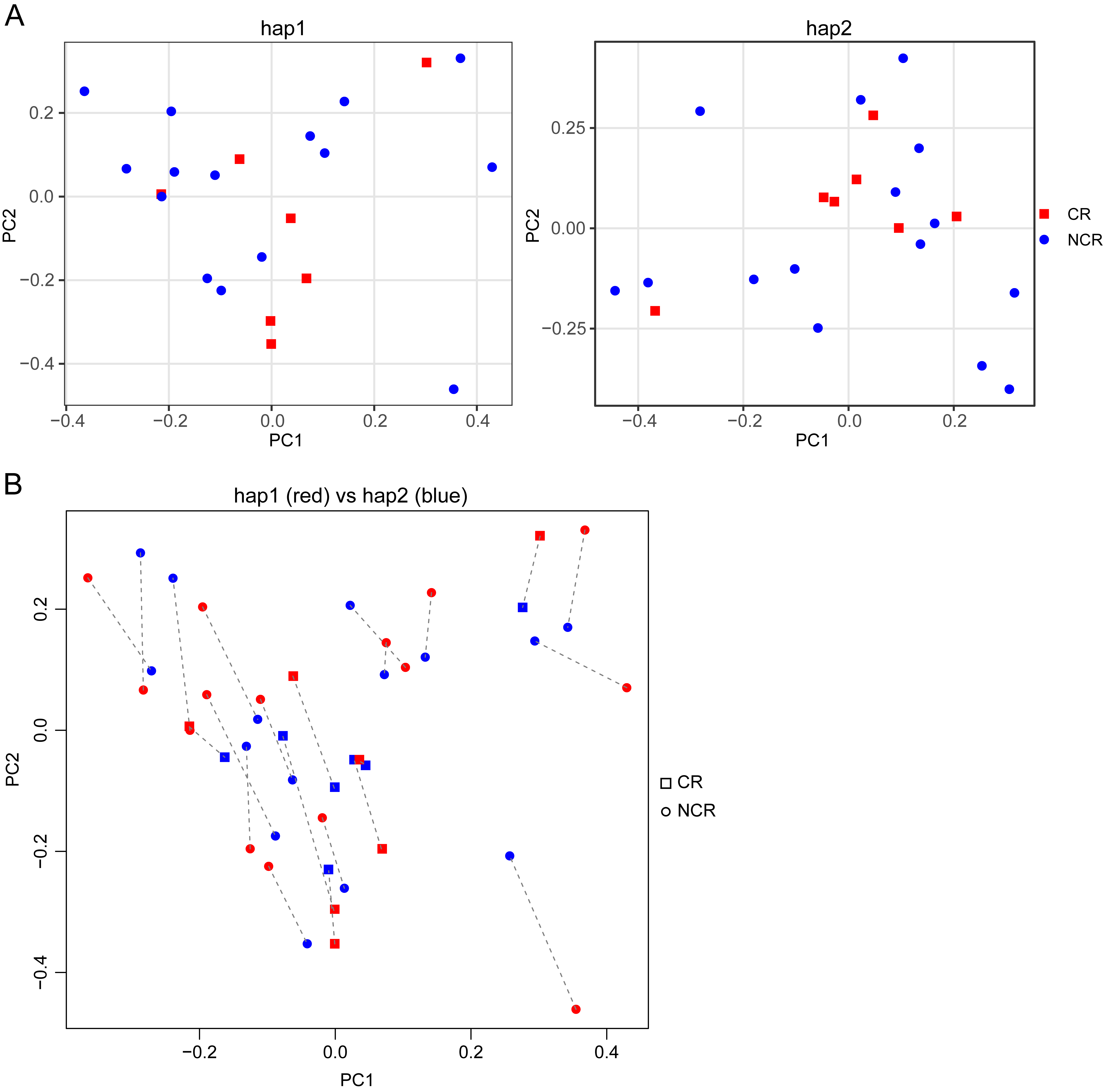


**Additional file 15.** **Principal component analysis (PCA) of the SNP distribution from all resquenced samples.** (A) The representation of PCA based on haplotype genome 1 and 2 separately. (B) The overlay representation of PCA based on haplotype genome 1 and 2.
